# Gram-negative bloodstream infections in pediatric patients: antimicrobial resistance and factors associated with mortality

**DOI:** 10.3389/fped.2026.1893910

**Published:** 2026-07-20

**Authors:** Asuman Akar, Mustafa Pişirici

**Affiliations:** 1 Department of Pediatrics, Division of Pediatric Infectious Diseases, Dicle University Faculty of Medicine, Diyarbakır, Türkiye; 2 Department of Microbiology, Dicle University Faculty of Medicine, Diyarbakır, Türkiye

**Keywords:** antimicrobial resistance, carbapenem resistance, ceftazidime–avibactam, gram-negative bacteria, intensive care unit, meropenem resistance, mortality, pediatric bloodstream infection

## Abstract

**Background:**

Gram-negative bloodstream infections (BSIs) are associated with substantial morbidity and mortality in pediatric patients, particularly among critically ill children. Increasing antimicrobial resistance among Gram-negative pathogens has further complicated empirical treatment strategies and limited therapeutic options. This study aimed to evaluate antimicrobial resistance patterns and factors associated with mortality in pediatric patients with Gram-negative BSIs.

**Methods:**

This retrospective cohort study included pediatric patients aged 0–18 years with laboratory-confirmed Gram-negative BSIs diagnosed between January 2022 and September 2025 at a tertiary care center. Demographic characteristics, invasive procedures, laboratory findings, antimicrobial susceptibility profiles, treatment-related variables, and clinical outcomes were analyzed. Factors associated with all-cause in-hospital mortality were evaluated using an exploratory multivariable logistic regression model.

**Results:**

A total of 252 pediatric patients were included. Klebsiella spp. (46.4%) and Acinetobacter spp. (28.6%) were the most frequently isolated pathogens. High resistance rates were observed for gentamicin (58.3%), ciprofloxacin (57.1%), imipenem (56.0%), and meropenem (48.4%). Ceftazidime–avibactam resistance was significantly associated with study year (*p* = 0.008). The overall all-cause in-hospital mortality rate was 31.7%. Prior meropenem exposure and antimicrobial treatment modification after blood culture collection were associated with higher mortality. In the revised multivariable model, central venous catheter use (adjusted OR=13.58, 95% CI: 5.02–36.60), thrombocytopenia (adjusted OR=4.82, 95% CI: 2.10–11.02), total parenteral nutrition (adjusted OR=4.83, 95% CI: 2.09–11.13), mechanical ventilation (adjusted OR=3.74, 95% CI: 1.41–9.97), and meropenem resistance (adjusted OR=3.00, 95% CI: 1.28–7.03) were independently associated with mortality.

**Conclusions:**

Gram-negative BSIs in pediatric patients were associated with substantial antimicrobial resistance and considerable all-cause in-hospital mortality. The increase in ceftazidime–avibactam resistance should be interpreted cautiously because molecular characterization was not performed. Continuous local surveillance, antimicrobial stewardship, and infection prevention strategies are important, particularly in high-risk neonatal and pediatric intensive care populations.

## 1 Introduction

Bloodstream infections (BSIs) remain among the most severe infectious diseases affecting pediatric patients, particularly neonates, infants, and children with underlying comorbidities (1). These infections are associated with substantial morbidity and mortality, prolonged hospitalization, and increased healthcare costs. Early diagnosis and timely initiation of appropriate antimicrobial therapy are therefore critical, as delays in effective treatment or inappropriate empirical antibiotic selection may rapidly lead to clinical deterioration, septic shock, and death.

In recent years, antimicrobial resistance among Gram-negative bacteria has emerged as a major global public health concern. This problem is particularly pronounced in tertiary care centers, where critically ill pediatric patients are frequently exposed to invasive procedures and broad-spectrum antibiotics (2). Gram-negative pathogens, especially Enterobacterales, Acinetobacter spp., and Pseudomonas spp., are increasingly associated with resistance to multiple antimicrobial agents (3, 4). The widespread and often inappropriate use of antibiotics has accelerated the emergence and dissemination of multidrug-resistant (MDR) organisms, significantly limiting available therapeutic options and contributing to the growing antimicrobial resistance (AMR) crisis (1, 5).

Carbapenem-resistant Gram-negative bacteria (CR-GNB) are considered critical priority pathogens because of their association with limited treatment options and high mortality rates (6). Resistance mechanisms such as carbapenemase production, efflux pump overexpression, and porin loss contribute to treatment failure and poor clinical outcomes (4, 7). In pediatric intensive care settings, these organisms are frequently associated with invasive interventions, prolonged hospitalization, and increased risk of transmission between patients, underscoring the importance of effective infection prevention and control practices.

Despite increasing awareness of antimicrobial resistance, important gaps remain in the pediatric literature. Much of the currently available evidence is derived from adult populations, while pediatric studies are often limited by relatively small sample sizes and heterogeneous patient groups (3). In addition, data integrating microbiological findings with clinical outcomes, particularly mortality, remain limited in many regions (1, 8). This lack of comprehensive data may complicate the optimization of empirical treatment strategies in high-risk pediatric patients.

Therefore, the present study aimed to evaluate antimicrobial resistance trends among Gram-negative pathogens causing bloodstream infections in pediatric patients and to identify factors associated with mortality. By analyzing microbiological, laboratory, and clinical findings from a tertiary care center, this study sought to provide clinically relevant data that may contribute to improved patient management, infection prevention strategies, and antimicrobial stewardship practices.

## 2 Materials and methods

### 2.1 Study design and setting

This retrospective cohort study was conducted at Dicle University Faculty of Medicine, a tertiary academic referral center in Türkiye. Pediatric patients diagnosed with Gram-negative bloodstream infections between January 1, 2022, and September 1, 2025, were evaluated. The study was reported in accordance with the Strengthening the Reporting of Observational Studies in Epidemiology (STROBE) guidelines for cohort studies.

### 2.2 Patient selection

Patients aged 0–18 years with laboratory-confirmed bloodstream infections caused by Gram-negative bacteria were eligible for inclusion. Bloodstream infection was defined as the isolation of Klebsiella spp. Acinetobacter spp., Escherichia coli, or Pseudomonas spp. from at least one blood culture in a patient with compatible clinical findings, including fever or hypothermia, clinical signs of sepsis, hemodynamic instability, elevated inflammatory markers, or initiation/modification of antimicrobial therapy by the treating physician.

Quantitative blood cultures were not performed during the study period. Because the organisms included in this study were clinically significant Gram-negative pathogens and not typical skin commensals, one positive blood culture was considered sufficient when accompanied by compatible clinical findings.

Only the first bloodstream infection episode for each patient was analyzed. For patients with repeated positive blood cultures, recurrent isolations from the same patient were excluded and were not considered new independent episodes in the present analysis. Polymicrobial bloodstream infection was defined as the isolation of more than one organism from blood cultures obtained during the same clinical episode. Polymicrobial episodes were excluded when pathogen-specific antimicrobial susceptibility and outcome attribution could not be reliably evaluated.

During the study period, 296 Gram-negative bloodstream infection episodes were initially evaluated. Twenty-six episodes were excluded because of polymicrobial bloodstream infection, and 18 patients were excluded because of incomplete clinical records or missing outcome data. The final study cohort included 252 pediatric patients.

The primary outcome of the study was all-cause in-hospital mortality following the index bloodstream infection episode. Infection-attributable mortality was not evaluated because of the retrospective design and the high burden of comorbidities and critical illness in the study population.

### 2.3 Clinical and laboratory data collection

Demographic, clinical, microbiological, and treatment-related data were obtained retrospectively from electronic hospital records. Variables evaluated included age, sex, admission unit, underlying diseases, intensive care unit admission, central venous catheter use, urinary catheterization, mechanical ventilation, total parenteral nutrition, antimicrobial susceptibility results, treatment-related variables, and in-hospital mortality.

Treatment-related variables included prior antimicrobial exposure, type of prior antibiotic therapy, duration of antibiotic use before the index blood culture, antimicrobial treatment modification after blood culture collection, and time from blood culture collection to treatment modification. Duration of prior antibiotic use was analyzed among patients with documented prior antibiotic exposure. Time to treatment modification was analyzed among patients who underwent antimicrobial treatment modification.

Laboratory parameters recorded on the day of bloodstream infection diagnosis included white blood cell count, absolute neutrophil count, hemoglobin level, platelet count, and C-reactive protein (CRP) level.

Anemia was defined as hemoglobin <12 g/dL, thrombocytopenia as platelet count <150,000/mm^3^, neutropenia as absolute neutrophil count <500/mm^3^, and leukocytosis as white blood cell count >15,000/mm^3^.

### 2.4 Microbiological methods

Venous blood samples were collected aseptically from patients with suspected bloodstream infection and inoculated into BACT/ALERT blood culture bottles (bioMérieux, France). Samples were processed in the microbiology laboratory according to routine laboratory procedures.

Bacterial identification was performed using automated identification systems, including VITEK 2 (bioMérieux, France) and/or matrix-assisted laser desorption ionization time-of-flight mass spectrometry (MALDI-TOF MS). Conventional biochemical methods were additionally used when necessary.

Antimicrobial susceptibility testing was performed using automated systems and/or disk diffusion methods according to routine laboratory workflow. Colistin susceptibility was determined using broth microdilution. Interpretation of susceptibility results was performed according to the Clinical and Laboratory Standards Institute (CLSI) and/or European Committee on Antimicrobial Susceptibility Testing (EUCAST) criteria applicable during the study period.

Because susceptibility testing for ceftriaxone, cefepime, ertapenem, and ceftazidime–avibactam was not routinely performed for Acinetobacter baumannii, analyses involving these antimicrobial agents were conducted after exclusion of Acinetobacter isolates.

Molecular characterization of antimicrobial resistance mechanisms was not routinely available and therefore was not performed. Multidrug-resistant, extensively drug-resistant, and pandrug-resistant isolates were classified according to standard international definitions based on nonsusceptibility across antimicrobial categories.

### 2.5 Statistical analysis

Statistical analyses were performed using IBM SPSS Statistics version 24.0 (IBM Corp., Chicago, IL, USA). Continuous variables were assessed for normality using the Shapiro–Wilk test. Normally distributed variables are presented as mean ± standard deviation (SD), whereas non-normally distributed variables are expressed as median and interquartile range (IQR). Categorical variables are presented as frequencies and percentages.

Comparisons between survivors and non-survivors were performed using Student's t-test or the Mann–Whitney U test for continuous variables, as appropriate. Categorical variables were compared using Pearson's chi-square test or Fisher's exact test. Temporal changes in antimicrobial resistance rates during the study period were analyzed using chi-square or Fisher's exact tests, as appropriate.

Treatment-related variables were analyzed descriptively and comparatively according to mortality status. To avoid a misleading zero-inflated distribution, the duration of prior antibiotic use was analyzed only among patients with documented prior antimicrobial exposure. Time from blood culture collection to antimicrobial treatment modification was analyzed only among patients who underwent treatment modification.

An exploratory multivariable logistic regression model was constructed to identify factors associated with all-cause in-hospital mortality. To reduce the risk of overfitting, the final model was revised as a parsimonious model including five clinically relevant variables: central venous catheter use, mechanical ventilation, total parenteral nutrition, meropenem resistance, and thrombocytopenia. Adjusted odds ratios (ORs) and 95% confidence intervals (CIs) were calculated.

Model fit and diagnostic performance were evaluated using deviance, Akaike information criterion (AIC), Bayesian information criterion (BIC), omnibus chi-square testing, Nagelkerke R^2^, receiver operating characteristic curve area under the curve (ROC-AUC), sensitivity, specificity, and accuracy. Multicollinearity among independent variables was assessed using variance inflation factor (VIF) analysis. Homoscedasticity testing was not applicable because logistic regression was used for a binary outcome rather than linear regression. The regression model was exploratory, was not externally validated, and was not intended to serve as a clinical prediction tool.

All statistical tests were two-tailed, and *p* < 0.05 was considered statistically significant.

### 2.6 Ethical approval

The study protocol was approved by the Ethics Committee of Dicle University Faculty of Medicine (approval number: 13; approval date: December 24, 2025). Ethics approval was obtained before data extraction and analysis. Due to the retrospective nature of the study, the requirement for informed consent was waived. All patient data were anonymized before analysis, and the study was conducted in accordance with the principles of the Declaration of Helsinki.

## 3 Results

The final study cohort included 252 pediatric patients. Of these, 114 patients (45.2%) were female and 138 (54.8%) were male. The mean age was 2.74 ± 4.5 years.

Regarding the distribution of patients across clinical settings, 111 patients (44.0%) were admitted to the pediatric intensive care unit, 107 patients (42.5%) to the neonatal intensive care unit, and 34 patients (13.5%) to general wards. Mortality differed according to admission unit. The highest mortality rate was observed in the neonatal intensive care unit, where 47 of 107 patients (43.9%) died, followed by the pediatric intensive care unit, where 33 of 111 patients (29.7%) died. No deaths occurred among the 34 patients treated in the general pediatric ward. Mortality was also higher among neonates aged ≤28 days than among older children [34/73 (46.6%) vs. 46/179 (25.7%), *p* = 0.002]. The demographic and clinical characteristics of the study population are summarized in Table 1.

**Table 1:** Demographic and clinical characteristics of the study population.

Characteristic	n (%)
Sex	
Male	138 (54.8)
Female	114 (45.2)
Age (years), mean ± SD	2.74 ± 4.5
Mortality	80 (31.7)
Hospital ward	
Neonatal intensive care unit	107 (42.5)
Pediatric intensive care unit	111 (44.0)
General ward	34 (13.5)
Underlying conditions	
Prematurity	91 (36.1)
Leukemia	30 (11.9)
Chronic lung disease	20 (7.9)
Metabolic disease	19 (7.5)
Burn	16 (6.3)
Intestinal atresia	15 (6.0)
Trauma-related hospitalization	12 (4.8)
Spinal muscular atrophy (SMA)	8 (3.2)
Immune disorder	7 (2.8)
Cerebral palsy	6 (2.4)
Cystic fibrosis	5 (2.0)
Congenital heart disease	4 (1.6)
Anal atresia	3 (1.2)
Esophageal atresia	3 (1.2)
Kidney stones	3 (1.2)
Other conditions*	3 (1.2)
Causative agents	
*Klebsiella spp.*	117 (46.4)
*Acinetobacter spp.*	72 (28.6)
*Escherichia coli*	36 (14.3)
*Pseudomonas spp.*	27 (10.7)
Interventions	
Central venous catheter	128 (50.8)
Mechanical ventilation	149 (59.1)
Total parenteral nutrition	99 (39.3)
Urinary catheter	90 (35.7)

Other conditions included diaphragmatic hernia, ventriculoperitoneal (VP) shunt infection, and liver transplantation. Patients may have had more than one underlying condition.

Pathogen-specific mortality was also evaluated. Mortality was highest among patients with Klebsiella spp. bloodstream infection [43/117 (36.8%)], followed by Acinetobacter spp. [22/72 (30.6%)], Escherichia coli [9/36 (25.0%)], and Pseudomonas spp. [6/27 (22.2%)]. The difference in mortality across pathogen groups was not statistically significant (*p* = 0.350).

Among all isolates, the highest resistance rate was observed for gentamicin (58.3%), followed by ciprofloxacin (57.1%), imipenem (56.0%), meropenem (48.4%), and amikacin (42.1%). Colistin demonstrated the lowest resistance rate (16.3%).

Because susceptibility testing for ceftriaxone, cefepime, ertapenem, and ceftazidime–avibactam was not routinely performed for *Acinetobacter baumannii*, analyses involving these antimicrobial agents were conducted after exclusion of *Acinetobacter* isolates. In this subgroup (*n* = 180), resistance was highest for ceftriaxone (78.9%), followed by cefepime (70.6%) and ertapenem (56.7%). Resistance to ceftazidime–avibactam remained relatively low (17.2%).

No significant temporal changes were observed in resistance to meropenem or colistin during the study period, whereas resistance to ceftazidime–avibactam increased significantly over time. Antimicrobial susceptibility profiles are summarized in Table 2.

**Table 2:** Antimicrobial susceptibility profiles of gram-negative bloodstream isolates.

Antibiotic	Susceptible, *n* (%)	Resistant, *n* (%)	Total (n)
Gentamicin	105 (41.7)	147 (58.3)	252
Ciprofloxacin	108 (42.9)	144 (57.1)	252
Imipenem	111 (44.0)	141 (56.0)	252
Meropenem	130 (51.6)	122 (48.4)	252
Amikacin	146 (57.9)	106 (42.1)	252
Colistin	211 (83.7)	41 (16.3)	252
Ceftriaxone*	38 (21.1)	142 (78.9)	180
Cefepime*	53 (29.4)	127 (70.6)	180
Ertapenem*	78 (43.3)	102 (56.7)	180
Ceftazidime–avibactam*	149 (82.8)	31 (17.2)	180

Analyses for ceftriaxone, cefepime, ertapenem, and ceftazidime–avibactam were performed after exclusion of Acinetobacter isolates.

Resistance co-occurrence patterns were additionally assessed. Meropenem plus colistin resistance was observed in 41 of 252 isolates (16.3%). Meropenem plus ceftazidime–avibactam resistance was observed in 31 of 180 isolates (17.2%), and combined meropenem, ceftazidime–avibactam, and colistin resistance was observed in 19 of 180 isolates (10.6%). Detailed resistance co-occurrence patterns are provided in Supplementary Table S2.

According to standard resistance category definitions, 78 of 252 isolates (31.0%) were classified as multidrug-resistant, 98 (38.9%) as extensively drug-resistant, and 21 (8.3%) as pandrug-resistant. Mortality increased across resistance categories: 7 of 55 patients (12.7%) with non-MDR isolates, 18 of 78 (23.1%) with MDR isolates, 42 of 98 (42.9%) with XDR isolates, and 13 of 21 (61.9%) with PDR isolates died during hospitalization (*p* < 0.001). The distribution of mortality according to MDR/XDR/PDR category is provided in Supplementary Table S3.

Pathogen-specific resistance profiles showed clinically relevant differences. Meropenem resistance was most frequent among Acinetobacter spp. isolates [58/72 (80.6%)], whereas colistin resistance was most frequent among Klebsiella spp. isolates [37/117 (31.6%)]. Ceftazidime–avibactam resistance was observed in 22 of 117 Klebsiella spp. isolates (18.8%), 6 of 27 Pseudomonas spp. isolates (22.2%), and 3 of 36 Escherichia coli isolates (8.3%). Detailed pathogen-specific resistance profiles are provided in Supplementary Table S4.

**Figure 1:**
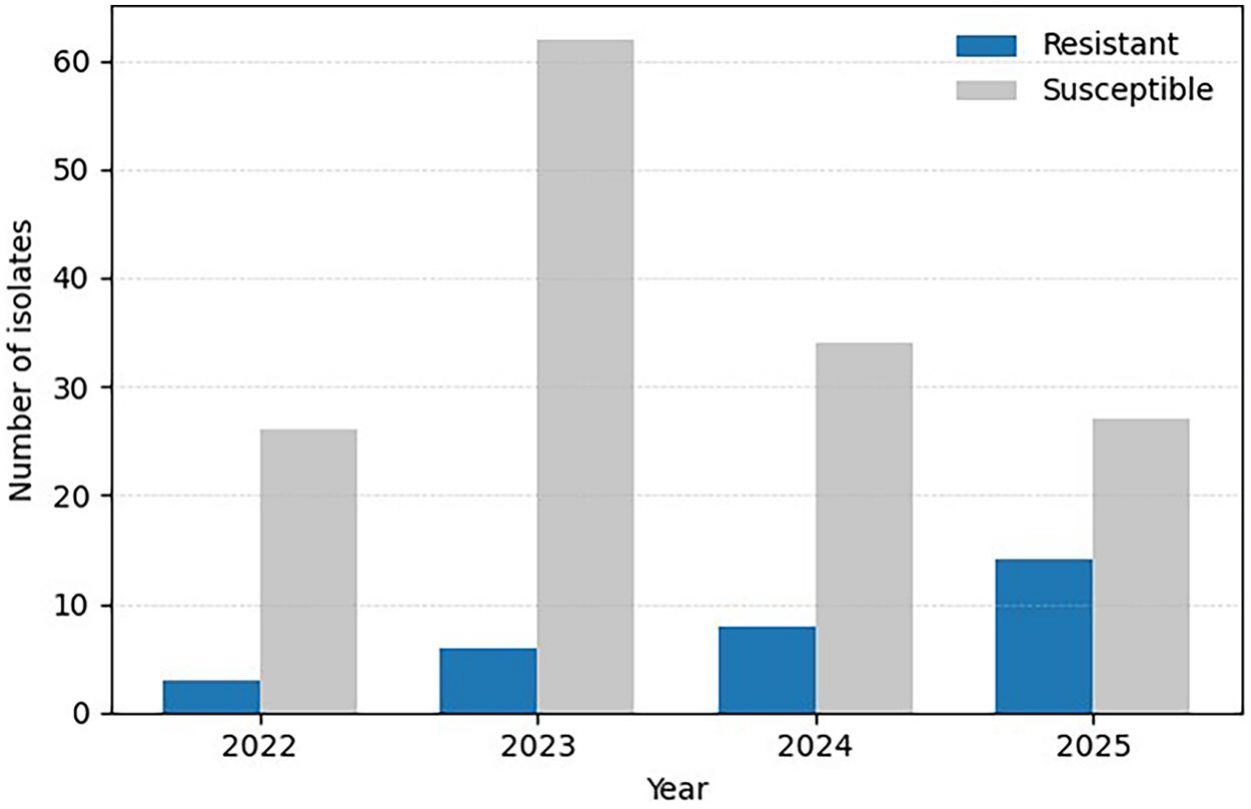
Temporal distribution of ceftazidime–avibactam-resistant and susceptible gram-negative bloodstream isolates between 2022 and 2025. A statistically significant association was observed between study year and resistance status (Fisher's exact test, *p* = 0.008).

Antimicrobial susceptibility to ceftazidime–avibactam over time is shown in Figure 1, and detailed annual counts are provided in Supplementary Table S1. Resistance was observed in 3 of 29 isolates (10.3%) in 2022, 6 of 68 isolates (8.8%) in 2023, 8 of 42 isolates (19.0%) in 2024, and 14 of 41 isolates (34.1%) in 2025. Overall, resistance was detected in 31 of 180 isolates (17.2%).

A statistically significant association was found between study year and ceftazidime–avibactam resistance (Fisher's exact test, *p* = 0.008). Resistance was highest in 2025.

**Figure 2:**
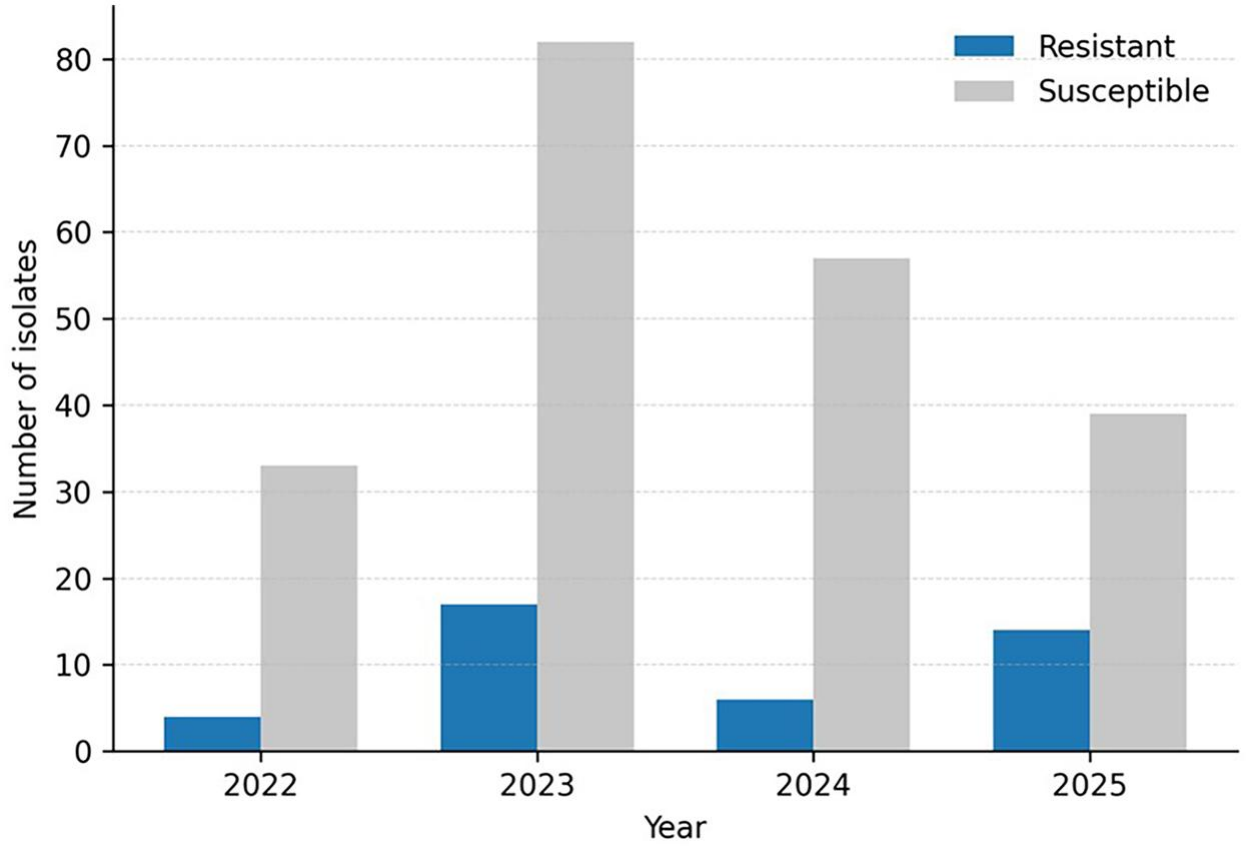
Temporal distribution of meropenem-resistant and susceptible gram-negative bloodstream isolates between 2022 and 2025. No statistically significant difference in resistance rates was observed across study years (*p* = 0.172).

The temporal distribution of meropenem susceptibility is shown in Figure 2. Meropenem resistance was observed in 12 of 37 isolates (32.4%) in 2022, 53 of 99 isolates (53.5%) in 2023, 32 of 63 isolates (50.8%) in 2024, and 25 of 53 isolates (47.2%) in 2025. Overall, 122 of 252 isolates (48.4%) were resistant, while 130 isolates (51.6%) were susceptible. Although resistance rates varied between years, no statistically significant difference was observed across the study years (Pearson's chi-square test, *p* = 0.172).

Figure 2. Temporal distribution of meropenem-resistant and susceptible Gram-negative bloodstream isolates between 2022 and 2025. No statistically significant difference in resistance rates was observed across study years (*p* = 0.172).

**Figure 3:**
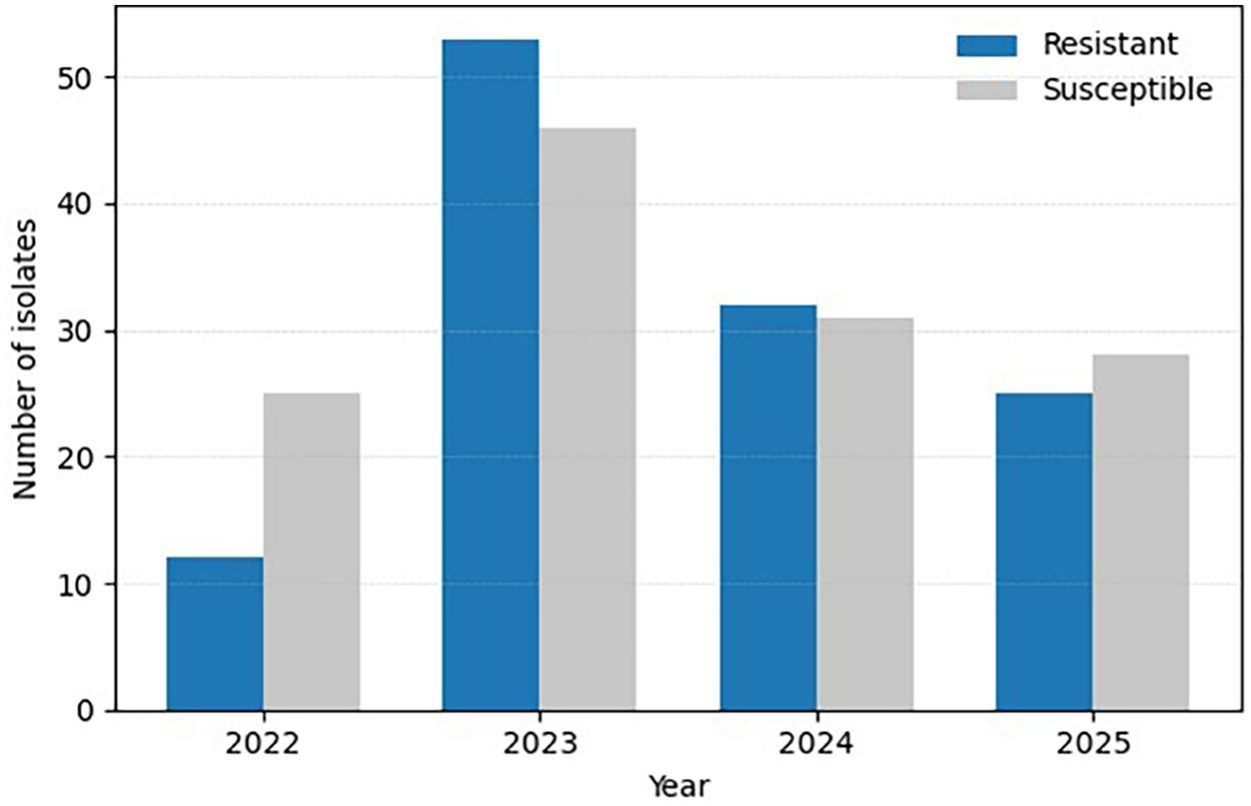
Temporal distribution of colistin-resistant and susceptible gram-negative bloodstream isolates between 2022 and 2025. No statistically significant difference in colistin resistance rates was observed across study years (*p* = 0.085).

The temporal distribution of colistin susceptibility is shown in Figure 3. Colistin resistance was observed in 4 of 37 isolates (10.8%) in 2022, 17 of 99 isolates (17.2%) in 2023, 6 of 63 isolates (9.5%) in 2024, and 14 of 53 isolates (26.4%) in 2025. Overall, resistance was detected in 41 of 252 isolates (16.3%), while 211 isolates (83.7%) were susceptible. Although resistance rates varied between years, no statistically significant difference was observed across the study years (Fisher's exact test, *p* = 0.085).

Treatment-related variables were analyzed according to in-hospital mortality status and are summarized in Tables 3, 4. Data on prior antimicrobial exposure were available for all patients. Overall, 140 patients had no documented prior antibiotic exposure, 64 had prior meropenem exposure, and 48 had prior piperacillin–tazobactam exposure. Mortality differed significantly according to prior antibiotic category: 41 of 140 patients (29.3%) without prior antibiotic exposure, 33 of 64 patients (51.6%) with prior meropenem exposure, and 6 of 48 patients (12.5%) with prior piperacillin–tazobactam exposure died during hospitalization (*p* < 0.001).

**Table 3:** Treatment-related variables according to in-hospital mortality status.

Variable	Survivors, *n* (%)	Non-survivors, *n* (%)	Total	*p*-value
Prior antibiotic exposure category				<0.001
No prior antibiotic exposure	99 (70.7)	41 (29.3)	140	
Prior meropenem exposure	31 (48.4)	33 (51.6)	64	
Prior piperacillin–tazobactam exposure	42 (87.5)	6 (12.5)	48	
Treatment modification after blood culture				<0.001
No	104 (80.0)	26 (20.0)	130	
Yes	68 (55.7)	54 (44.3)	122	

Values are presented as *n* (% within row). Comparisons were performed using the chi-square test or Fisher's exact test, as appropriate.

**Table 4:** Duration of prior antibiotic exposure and time to treatment modification according to in-hospital mortality status.

Variable	Survivors, median (IQR)	Non-survivors, median (IQR)	*p*-value
Duration of antibiotic use before index blood culture among patients with prior antibiotic exposure, days	21 (14–42)	21 (17.5–28)	0.552
Time from blood culture collection to treatment modification among patients who underwent treatment modification, hours	72 (48–80.5)	75.5 (48–95)	0.033

Values are presented as median and interquartile range (IQR). Duration of prior antibiotic use was analyzed only among patients with documented prior antibiotic exposure. Time to treatment modification was analyzed only among patients who underwent antimicrobial treatment modification. Comparisons were performed using the Mann–Whitney U test.

Antimicrobial treatment modification after blood culture collection was performed in 122 of 252 patients (48.4%). Mortality was significantly higher among patients who underwent treatment modification than among those who did not [54/122 (44.3%) vs. 26/130 (20.0%); *p* < 0.001]. Prior antibiotic exposure category and antimicrobial treatment modification were significantly associated with mortality (Table 3).

Among patients with documented prior antibiotic exposure, the duration of antibiotic use before the index blood culture did not differ significantly between survivors and non-survivors [median (IQR): 21 (14–42) vs. 21 (17.5–28) days; *p* = 0.552]. Among patients who underwent antimicrobial treatment modification, the median time from blood culture collection to treatment modification was longer in non-survivors than in survivors [75.5 (48–95) vs. 72 (48–80.5) hours; *p* = 0.033] (Table 4).

These findings should be interpreted cautiously, as treatment modification may reflect resistant infection, inadequate initial antimicrobial coverage, or greater illness severity rather than a direct causal effect of treatment modification itself.

Comparisons between survivors and non-survivors are presented in Table 5. Non-survivors were significantly younger and had significantly lower platelet counts than survivors, whereas C-reactive protein levels were significantly higher in non-survivors (all *p* < 0.05). No significant differences were observed between the groups in white blood cell count, absolute neutrophil count, or hemoglobin level.

**Table 5:** Comparison of selected clinical and laboratory parameters between survivors and non-survivors.

Variable	Non-survivors (*n* = 80), median (IQR)	Survivors (*n* = 172), median (IQR)	*p*-value
White blood cell count (/mm^3^)	12,392 (7,160–16,390)	12,407 (3,750–18,550)	0.335
Neutrophil count (/mm^3^)	7,694 (3,200–9,818)	8,328 (1,988–11,250)	0.861
Hemoglobin (g/dL)	10.56 (8.9–12.0)	10.76 (9.3–12.0)	0.751
Platelet count (/mm^3^)	136,716 (34,000–199,750)	270,976 (152,250–376,250)	<0.001
CRP (mg/L)	140.9 (49.5–211.0)	93.9 (21.8–139.0)	0.004
Age (years)	0.18 (0.03–2.11)	0.87 (0.10–3.91)	0.002

Values are presented as median and interquartile range (IQR). CRP, C-reactive protein. Comparisons were performed using the Mann–Whitney U test.

To reduce the risk of overfitting, the multivariable logistic regression model was revised as a parsimonious exploratory model including five clinically relevant variables associated with all-cause in-hospital mortality. The model included all 252 patients and 80 mortality events, corresponding to 16 events per variable. The overall model was statistically significant (*χ*^2^ = 151, df = 5, *p* < 0.001), with a Nagelkerke R^2^ value of 0.632.

In the revised model, central venous catheter use, total parenteral nutrition, thrombocytopenia, mechanical ventilation, and meropenem resistance were independently associated with all-cause in-hospital mortality (Table 6). Central venous catheter use showed the strongest association with mortality (adjusted OR=13.58, 95% CI: 5.02–36.60, *p* < 0.001), followed by total parenteral nutrition (adjusted OR=4.83, 95% CI: 2.09–11.13, *p* < 0.001), thrombocytopenia (adjusted OR=4.82, 95% CI: 2.10–11.02, *p* < 0.001), mechanical ventilation (adjusted OR=3.74, 95% CI: 1.41–9.97, *p* = 0.008), and meropenem resistance (adjusted OR=3.00, 95% CI: 1.28–7.03, *p* = 0.012).

**Table 6:** Multivariable logistic regression analysis of factors associated with all-cause in-hospital mortality.

Variable	Estimate	Wald z	*p*-value	Adjusted OR	95% CI for OR
Central venous catheter use	2.61	5.14	<0.001	13.58	5.02–36.60
Mechanical ventilation	1.32	2.65	0.008	3.74	1.41–9.97
Total parenteral nutrition	1.57	3.69	<0.001	4.83	2.09–11.13
Meropenem resistance	1.10	2.52	0.012	3.00	1.28–7.03
Thrombocytopenia	1.57	3.72	<0.001	4.82	2.10–11.02

OR, odds ratio; CI, confidence interval. The reference category for each variable was the absence of the corresponding factor. Thrombocytopenia was entered into the model as a dichotomous variable, defined as platelet count <150,000/mm^3^. The model included all 252 patients and 80 mortality events.

The model demonstrated good discrimination within the study cohort, with an ROC-AUC value of 0.924. Using a cut-off value of 0.5, the model showed an accuracy of 85.7%, specificity of 89.5%, and sensitivity of 77.5%. However, the model was exploratory, was not externally validated, and should not be interpreted as a clinical prediction tool. Collinearity diagnostics and model fit/performance measures are provided in Supplementary Tables S5 and S6.

**Figure 4:**
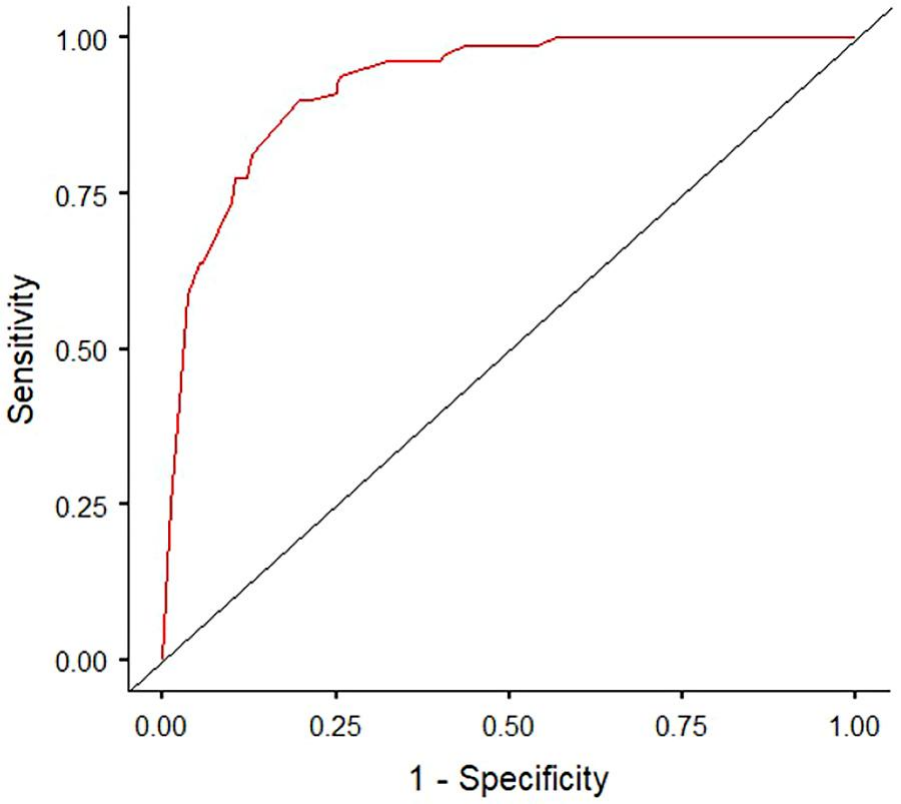
Receiver operating characteristic (ROC) curve of the revised exploratory multivariable logistic regression model for all-cause in-hospital mortality. The model demonstrated good discrimination within the study cohort, with an ROC-AUC value of 0.924.

The receiver operating characteristic (ROC) curve of the revised exploratory multivariable logistic regression model is shown in Figure 4.

## 4 Discussion

The management of Gram-negative bloodstream infections in children remains challenging, mainly because of the increasing burden of antimicrobial resistance. Carbapenem-resistant Gram-negative infections are frequently reported in high-risk units, including neonatal and pediatric intensive care units, where invasive procedures and broad-spectrum antibiotic exposure are common (9–12). In our cohort, most patients were treated in intensive care settings, which reflects the severity of illness in the study population. Therefore, the findings of the present study should be interpreted primarily in the context of a high-risk tertiary care cohort with a predominance of neonatal and pediatric intensive care patients.

The distribution of pathogens was broadly consistent with previous pediatric studies, in which Escherichia coli, Klebsiella spp., and Enterobacter spp. have been reported among the leading causes of Gram-negative bloodstream infections (13–15). In the present study, Klebsiella spp. was the most common pathogen, followed by Acinetobacter spp., Escherichia coli, and Pseudomonas spp. The relatively high proportion of Acinetobacter spp. is clinically relevant, particularly in intensive care units, where this organism can persist in the hospital environment and acquire resistance rapidly.

Antimicrobial resistance was common in our cohort. Resistance rates to gentamicin, ciprofloxacin, imipenem, and meropenem were high, indicating that several commonly used agents may have limited reliability for empirical treatment in this setting. Similar increases in resistance among Gram-negative pathogens, particularly to aminoglycosides, fluoroquinolones, and cephalosporins, have been reported globally (16). Carbapenem-resistant Gram-negative infections are now an important clinical problem, especially in tertiary care centers and low- and middle-income countries (17–20). As carbapenems are often used for severe Gram-negative infections, resistance to these agents can substantially restrict treatment choices and may require older or combination antimicrobial regimens (21).

The additional MDR/XDR/PDR classification and resistance co-occurrence analyses further demonstrate the burden of antimicrobial resistance in this cohort. Mortality increased across resistance categories, from non-MDR to PDR isolates, suggesting that broader resistance phenotypes may be markers of more difficult-to-treat infections and more severe clinical courses. Co-resistance involving meropenem, colistin, and ceftazidime–avibactam is particularly concerning because these agents are often considered in the management of severe resistant Gram-negative infections. Nevertheless, these findings should be interpreted within the limitations of a single-center retrospective study without molecular resistance characterization.

Gram-negative pathogens such as Klebsiella pneumoniae, Escherichia coli, Acinetobacter baumannii, and Pseudomonas aeruginosa can develop resistance through several mechanisms, including *β*-lactamase production, efflux pump activity, porin alterations, and carbapenemase production (22, 23). In the present study, ceftazidime–avibactam resistance increased significantly between 2022 and 2025. However, this finding should be interpreted cautiously because the annual sample sizes were limited and molecular characterization of resistance mechanisms was not performed. Therefore, the mechanisms underlying ceftazidime–avibactam resistance could not be determined. The observed increase may reflect changes in local resistance epidemiology, but molecular studies are required to confirm the underlying mechanisms (24–27).

Resistant infections may be associated with delayed effective antimicrobial therapy, increased risk of treatment failure, prolonged hospitalization, and poor outcomes (28, 29). The overall all-cause in-hospital mortality rate in our cohort was 31.7%, which is high but comparable with reports from high-risk pediatric populations with multidrug-resistant Gram-negative infections (20, 33, 34). However, mortality in this study should not be interpreted as infection-attributable mortality. Many patients had substantial comorbidities, invasive device exposure, and critical illness; therefore, death may have resulted from a combination of bloodstream infection, underlying disease severity, and treatment-related factors. Non-survivors were younger and had lower platelet counts and higher C-reactive protein levels than survivors, suggesting a greater inflammatory burden and more severe clinical course.

In the revised exploratory multivariable logistic regression model, central venous catheter use, total parenteral nutrition, thrombocytopenia, mechanical ventilation, and meropenem resistance were independently associated with all-cause in-hospital mortality. These variables should be interpreted as factors associated with mortality rather than as direct causal determinants. Central venous catheter use, total parenteral nutrition, and mechanical ventilation may reflect both invasive treatment exposure and greater severity of illness or prolonged intensive care support (30–32). Because validated severity-of-illness scores such as PRISM, PIM, or neonatal severity scores were not available, residual confounding by disease severity cannot be excluded.

Meropenem resistance remained associated with mortality in the revised model. This association may reflect limited therapeutic options, delayed effective antimicrobial coverage, higher baseline severity, or infection caused by more resistant organisms. However, because standardized data on empirical treatment appropriateness and time to first active therapy were not fully available, this association should be interpreted cautiously (35).

The added treatment-related analyses provide additional context. Prior meropenem exposure was associated with higher mortality, whereas the duration of antibiotic use before the index blood culture did not differ significantly between survivors and non-survivors among patients with documented prior antibiotic exposure. Antimicrobial treatment modification after blood culture collection was also associated with higher mortality, and the time from blood culture collection to treatment modification was longer among non-survivors. These findings may suggest that patients requiring treatment modification represented a clinically more complex group, possibly with resistant infection, inadequate initial coverage, or greater illness severity. Therefore, treatment modification should not be interpreted as a direct cause of mortality.

Our findings support the need for continuous local surveillance of antimicrobial resistance patterns. Local resistance data are essential for guiding empirical treatment decisions, particularly in intensive care units where resistant pathogens are common. In parallel, antimicrobial stewardship and infection prevention measures, including hand hygiene and device-related care bundles, remain key components of reducing the burden of resistant Gram-negative infections in pediatric patients (36, 37).

Overall, this study suggests that Gram-negative bloodstream infections in pediatric patients treated in a tertiary care setting are associated with substantial antimicrobial resistance and high all-cause in-hospital mortality. The increasing ceftazidime–avibactam resistance and the association between meropenem resistance and mortality are clinically important observations. These findings support the importance of continuous local resistance surveillance, antimicrobial stewardship, and infection prevention strategies, particularly in high-risk neonatal and pediatric intensive care populations.

### 4.1 Limitations

This study has several limitations. First, its retrospective single-center design may limit the generalizability of the findings. Second, the high proportion of neonatal and pediatric intensive care patients indicates a high-severity cohort and may limit applicability to general ward pediatric populations. Third, exclusion of 26 polymicrobial bloodstream infection episodes and 18 patients with incomplete clinical records or missing outcome data may have introduced selection bias. Fourth, only the first bloodstream infection episode per patient was analyzed, and recurrent episodes were not evaluated. Fifth, the primary outcome was all-cause in-hospital mortality rather than infection-attributable mortality; therefore, deaths may have been influenced by underlying comorbidities, critical illness, and treatment-related factors in addition to bloodstream infection itself. Sixth, validated severity-of-illness scores such as PRISM, PIM, or neonatal severity scores were not available; therefore, residual confounding by disease severity is likely. Seventh, although data on prior antimicrobial exposure, antibiotic type, duration of prior antibiotic use, antimicrobial treatment modification, and time to treatment modification were analyzed, standardized data on empirical treatment appropriateness and time to first active therapy were not fully available. Eighth, molecular characterization of antimicrobial resistance mechanisms was not performed, limiting interpretation of the mechanisms underlying ceftazidime–avibactam and carbapenem resistance. Finally, although the multivariable model was revised to a more parsimonious exploratory model with 16 events per variable, potential model overfitting and lack of external validation cannot be fully excluded.

## 5 Conclusion

Gram-negative bloodstream infections in pediatric patients were associated with substantial antimicrobial resistance and high all-cause in-hospital mortality in this tertiary care cohort. Ceftazidime–avibactam resistance increased significantly during the study period, although this finding should be interpreted cautiously because molecular characterization was not performed and annual sample sizes were limited. Central venous catheter use, total parenteral nutrition, thrombocytopenia, mechanical ventilation, and meropenem resistance were independently associated with all-cause in-hospital mortality in the revised exploratory multivariable model. These findings support the importance of continuous local resistance surveillance, antimicrobial stewardship, and infection prevention strategies, particularly in high-risk neonatal and pediatric intensive care populations.

## Data Availability

The raw data supporting the conclusions of this article will be made available by the authors, without undue reservation.
