# Supplementary material for: Gram-negative bloodstream infections in pediatric patients: antimicrobial resistance and factors associated with mortality
Source: Front Pediatr. 2026 Jul 20;14:1893910. doi: 10.3389/fped.2026.1893910 (PMC13429780; doi:10.3389/fped.2026.1893910)
Supplement: Supplementary file 1 [file Table1.docx]

**Supplementary Table S1.** Temporal distribution of ceftazidime–avibactam susceptibility

| **Year** | **Resistant, n (%)** | **Susceptible, n (%)** | **Total** |
| --- | --- | --- | --- |
| 2022 | 3 (10.3) | 26 (89.7) | 29 |
| 2023 | 6 (8.8) | 62 (91.2) | 68 |
| 2024 | 8 (19.0) | 34 (81.0) | 42 |
| 2025 | 14 (34.1) | 27 (65.9) | 41 |
| **Total** | **31 (17.2)** | **149 (82.8)** | **180** |

**Supplementary Table S2**. Resistance co-occurrence patterns

| **Resistance pattern** | **Resistant isolates, n (%)** | **Total tested** |
| --- | --- | --- |
| Meropenem plus colistin resistance | 41 (16.3) | 252 |
| Meropenem plus ceftazidime–avibactam resistance | 31 (17.2) | 180 |
| Meropenem plus ceftazidime–avibactam plus colistin resistance | 19 (10.6) | 180 |

* Ceftazidime–avibactam-based analyses were performed after exclusion of Acinetobacter spp. isolates because ceftazidime–avibactam susceptibility was not routinely tested for Acinetobacter spp.

**Supplementary Table S3.** MDR, XDR, and PDR distribution according to in-hospital mortality status

| **Resistance category** | **Survivors, n (%)** | **Non-survivors, n (%)** | **Total** | **Mortality rate** | **p-value** |
| --- | --- | --- | --- | --- | --- |
| Non-MDR | 48 (87.3) | 7 (12.7) | 55 | 12.7% | <0.001 |
| MDR | 60 (76.9) | 18 (23.1) | 78 | 23.1% |  |
| XDR | 56 (57.1) | 42 (42.9) | 98 | 42.9% |  |
| PDR | 8 (38.1) | 13 (61.9) | 21 | 61.9% |  |

* MDR, multidrug-resistant; XDR, extensively drug-resistant; PDR, pandrug-resistant. Values are presented as n (% within row). The p-value represents comparison across resistance categories.

**Supplementary Table S4**. Pathogen-specific selected resistance profiles

| **Pathogen** | **Meropenem resistance** | **Colistin resistance** | **Ceftazidime–avibactam resistance** | **Ertapenem resistance** | **Ceftriaxone resistance** | **Cefepime resistance** |
| --- | --- | --- | --- | --- | --- | --- |
| Acinetobacter spp. | 58/72 (80.6) | 3/72 (4.2) | NA | NA | NA | NA |
| Escherichia coli | 8/36 (22.2) | 0/36 (0.0) | 3/36 (8.3) | 10/36 (27.8) | 25/36 (69.4) | 14/36 (38.9) |
| Klebsiella spp. | 48/117 (41.0) | 37/117 (31.6) | 22/117 (18.8) | 77/117 (65.8) | 90/117 (76.9) | 86/117 (73.5) |
| Pseudomonas spp. | 8/27 (29.6) | 0/27 (0.0) | 6/27 (22.2) | NA | 27/27 (100.0) | 27/27 (100.0) |

* Values are presented as resistant isolates/total tested (%). NA indicates that susceptibility testing was not routinely performed or was not applicable for that pathogen-antimicrobial combination.

**Supplementary Table S5.** Collinearity diagnostics for variables included in the multivariable logistic regression model

| **Variable** | **VIF** | **Tolerance** |
| --- | --- | --- |
| Central venous catheter use | 1.02 | 0.983 |
| Mechanical ventilation | 1.10 | 0.911 |
| Total parenteral nutrition | 1.17 | 0.854 |
| Meropenem resistance | 1.13 | 0.887 |
| Thrombocytopenia | 1.04 | 0.958 |

* VIF, variance inflation factor. All VIF values were below 2, indicating no relevant multicollinearity.

**Supplementary Table S6.** Model fit and diagnostic performance of the multivariable logistic regression model

| **Measure** | **Value** |
| --- | --- |
| Deviance | 164 |
| AIC | 176 |
| BIC | 197 |
| Nagelkerke R² | 0.632 |
| Overall model χ² | 151 |
| df | 5 |
| p-value | <0.001 |
| ROC-AUC | 0.924 |
| Accuracy | 85.7% |
| Sensitivity | 77.5% |
| Specificity | 89.5% |

* AIC, Akaike information criterion; BIC, Bayesian information criterion; ROC-AUC, receiver operating characteristic curve area under the curve. The model was exploratory, was not externally validated, and should not be interpreted as a clinical prediction tool.
